# Dietary Intake and Arsenic Methylation in a U.S. Population

**DOI:** 10.1289/ehp.7907

**Published:** 2005-05-10

**Authors:** Craig Steinmaus, Kenichi Carrigan, Dave Kalman, Raja Atallah, Yan Yuan, Allan H. Smith

**Affiliations:** 1 Arsenic Health Effects Research Program, School of Public Health, University of California, Berkeley, California, USA; 2 Division of Occupational and Environmental Medicine, University of California, San Francisco, California, USA; 3 School of Public Health and Community Medicine, University of Washington, Seattle, Washington, USA

**Keywords:** arsenic, drinking water, environmental health, metabolism, nutritional susceptibility

## Abstract

Millions of people worldwide are exposed to arsenic-contaminated drinking water, and ingestion of inorganic arsenic (InAs) has been associated with increased risks of cancer. The primary metabolic pathway of ingested InAs is methylation to monomethyl arsenic (MMA) and dimethyl arsenic (DMA). However, people vary greatly in the degree to which they methylate InAs, and recent evidence suggests that those who excrete high proportions of ingested arsenic as MMA are more susceptible than others to arsenic-caused cancer. To date, little is known about the factors that determine interindividual differences in arsenic methylation. In this study, we assessed the effect of diet on arsenic metabolism by measuring dietary intakes and urinary arsenic methylation patterns in 87 subjects from two arsenic-exposed regions in the western United States. Subjects in the lower quartile of protein intake excreted a higher proportion of ingested InAs as MMA (14.6 vs. 11.6%; *p* = 0.01) and a lower proportion as DMA (72.3 vs. 77.0%; *p* = 0.01) than did subjects in the upper quartile of protein intake. Subjects in the lower quartile of iron, zinc, and niacin intake also had higher urinary percent MMA and lower percent DMA levels than did subjects with higher intakes of these nutrients. These associations were also seen in multivariate regression analyses adjusted for age, sex, smoking, and total urinary arsenic. Given the previously reported links between high percent MMA and increased cancer risks, these findings are consistent with the theory that people with diets deficient in protein and other nutrients are more susceptible than others to arsenic-caused cancer.

Inorganic arsenic (InAs) occurs naturally in the groundwater of many parts of the world, and millions of people worldwide are exposed to drinking water containing this known carcinogen (Nordstrom 2002). Ingested arsenic causes cancers of the skin, bladder, and lung and has been associated with cancers of other organs [National Research Council (NRC) 1999, 2001]. The estimated risks associated with these exposures may be quite high. According to a subcommittee of the NRC, the cancer risks associated with lifetime exposures at the new U.S. standard of 10 μg/L may be as high as 1 in 300 (NRC 1999, 2001). The U.S. drinking water standards for other carcinogens have been set at levels associated with cancer risks that are about 30–3,000 times lower (Smith et al. 2002). Importantly, the new U.S. standard for arsenic applies only to public water systems. Approximately 15% of the U.S.population obtain their water from private wells (U.S. Geological Survey 2004), and arsenic concentrations > 10 μg/L have been documented in private wells throughout the United States (Ayotte et al. 2003; Steinmaus et al. 2003; Welch et al. 1999).

The primary metabolic pathway of ingested InAs in humans is methylation (Gebel 2002; Styblo et al. 2002; Vahter 2002). Ingested InAs is first methylated to monomethylarsonic acid (MMA5), which is reduced to monomethylarsonous acid (MMA3). MMA3 is then methylated to dimethylarsinic acid (DMA5), which is reduced to dimethylarsinous acid (DMA3). In humans, this process is not complete, and some arsenic remains as either InAs or MMA. Typically, ingested InAs is excreted as 10–20% InAs, 10–15% monomethyl arsenic (MMA), and 60–75% dimethyl arsenic (DMA) (Hopenhayn-Rich et al. 1993). However, large interindividual variations exist.

Until recently, methylation was thought to be primarily a detoxification pathway. This was based on the finding that MMA5 and DMA5—the most common forms of MMA and DMA found in exposed humans—are more readily excreted and less toxic than is InAs (Buchet et al. 1981; Gebel 2002; Hughes and Kenyon 1998; Moore et al. 1997). The trivalent forms of MMA and DMA are rapidly oxidized in urine and therefore are difficult to measure in human epidemiologic studies. Recently, however, methods have been developed to stabilize and measure MMA3 and DMA3 in urine, and these metabolites have been identified in urine samples from arsenic-exposed humans (Aposhian et al. 2000; Del Razo et al. 2001; Le et al. 2000; Mandal et al. 2001; Wang et al. 2004). Laboratory studies have shown that the trivalent forms of MMA and DMA are much more toxic than the pentavalent forms, and *in vitro* evidence suggests that MMA3 in particular may be more toxic than trivalent inorganic arsenic (InAs3) (Cullen et al. 1989; Lin et al. 1999, 2001; Mass et al. 2001; Petrick et al. 2000; Styblo et al. 1997, 1999, 2000). These findings suggest that methylation may not be strictly a detoxification pathway.

In fact, several epidemiologic studies have reported associations between elevated urinary proportions of MMA and increased risks of arsenic-associated health effects. In four studies from arsenic-exposed regions in Taiwan, subjects who excreted high proportions of urinary arsenic as MMA (percent MMA) or had high urinary MMA:DMA ratios had skin and bladder cancer odds ratios (OR) that were two to five times higher than did subjects who excreted low proportions of urinary MMA or had low MMA:DMA ratios (Chen et al. 2003a, 2003b; Hsueh et al. 1997; Yu et al. 2000). Associations between high levels of urinary percent MMA and increased bladder cancer risks were also found in studies on arsenic-exposed populations in the United States and Argentina (Steinmaus et al. 2004). Other studies have reported links between elevated urinary percent MMA or an elevated MMA:DMA ratio and increased risks of arsenic-caused skin lesions and increased rates of chromosomal aberrations (Del Razo et al. 1997; Maki-Paakkanen et al. 1998). The consistency of these associations, across different studies and different study populations, provides fairly strong evidence that individual differences in arsenic methylation patterns, and the environmental or genetic factors that cause these differences, play an important role in susceptibility to arsenic-caused disease.

To date, the environmental or genetic factors that control arsenic methylation are largely unknown. This is the first study to report on the impact of dietary protein, zinc, iron, thiamine, and several other potentially important macro- and micronutrients on arsenic methylation in humans.

## Materials and Methods

Subjects were recruited from among residents of six counties in western Nevada and Kings County in California. These areas contain the cities of Hanford, California, and Fallon, Nevada, the largest populations in the United States with historically high water arsenic levels (Steinmaus et al. 2003). Historically, arsenic levels in the drinking water supplies in these cities had been near 100 μg/L, although levels in Hanford have dropped to < 50 μg/L over the past 15 years because of the development of new wells. In Fallon, an arsenic treatment plant has recently been installed to meet the new U.S. arsenic standard of 10 μg/L. Most other cities in the study area have public water supplies with arsenic levels < 20 μg/L. Approximately 20% of the study area residents obtain water from private wells where arsenic levels range from below detection to > 1,000 μg/L.

Most of the study subjects were recruited from the participants of a case–control study of bladder cancer and arsenic exposure (Steinmaus et al. 2003). Subjects with bladder cancer were obtained from state cancer registries and from local hospitals and physicians. Control subjects were selected through random digit dialing (RDD) and from randomly selected lists provided by the Health Care Financing Administration. Further details on the selection of subjects for the case–control study are described elsewhere (Steinmaus et al. 2003). All participants who had lived in the cities of Fallon or Hanford or the nearby surrounding areas for at least the 1 year preceding recruitment were invited to participate in the methylation study. Because the bladder cancer case–control study included mostly men > 60 years of age, 15 additional subjects, mostly females and subjects < 60 years of age (average age = 48, 53% female), were recruited for this study using RDD. These 15 subjects were recruited during the same time period as the controls in the larger case–control study, and the same methods were used to assess their diets and urine metabolites. Removing these subjects had little impact on our results.

Most arsenic ingested by humans is excreted in urine, and the relative distribution of arsenic metabolites in urine is commonly used as a biomarker of arsenic methylation patterns (NRC 1999). Two to three urine samples were collected from each participant over a 1-year period. Subjects were given screw-top polypropylene containers and asked to give a midstream sample of the first morning void. A previous study has shown strong correlations in arsenic excretion between single first-morning samples and samples collected over 24 hr (Calderon et al. 1999). Samples were then transported on ice to the field laboratory each day where they were kept frozen at −20°C. Urine samples were transported overnight on dry ice to the University of Washington, Seattle, for analysis. This study was approved by the University of California, Berkeley, Committee for Protection of Human Subjects.

The urinary concentrations of arsenic were measured using hydride generation atomic absorption spectroscopy (Crecelius 1978). Briefly, inorganic arsenic (InAs3 and InAs5), MMA, and DMA were reduced to the corresponding arsine in a batch reactor using sodium borohydride in 5-mL samples. The volatile reduction products (arsine, methyl arsine, and dimethylarsine) were removed by sparging with helium. Entrained arsines were concentrated in a chromosorb-filled cryogenic trap in liquid nitrogen temperatures until all arsine-forming arsenic in the sample had reacted. The cryotrap was then allowed to warm, and the collected arsines were separated on the basis of differential volatilization. We detected the separated volatile arsenic species using atomic absorption spectroscopy with a hydrogen microburner combustion cell to convert arsines to elemental arsenic (PerkinElmer, Inc., Wellesley, MA). To prevent interference by certain compounds (Del Razo et al. 1999), each urine sample was acidified with 2 M HCl and allowed to sit for at least 4 hr. Total arsenic was determined by flow injection analysis/atomic fluorescence spectrometry (PS Analytical, Inc., Orpington, Kent, UK), and this result was compared with the sum of the species detected. If a significant amount of arsenic remained undetected, additional digestion or assay for arsenobetaine was performed. Detection limits for InAs, MMA, and DMA were 0.5, 1.0, and 2.0 μg/L, respectively. Concentrations below the detection limit were set at one half the detection limit. The MMA and DMA measured in this study were in the pentavalent forms. The trivalent forms, MMA3 and DMA3, are rapidly oxidized to MMA5 and DMA5 during storage (Del Razo et al. 2001). Most samples in this study were frozen for 2–6 weeks before analysis. We analyzed a subsample of urine specimens for MMA3 and DMA3 but found no MMA3 and only trace amounts of DMA3.

We used the National Cancer Institute (NCI)’s Health Habits and History Questionnaire (HHHQ) (Block et al. 1986) to collect dietary information from each subject. The full HHHQ was administered over the telephone by trained study personnel. Subjects were asked about their typical frequency and portion sizes for each food item over the preceding year because our *a priori* hypothesis was that relatively long-term dietary patterns influence arsenic methylation. We assessed nutrient intake by multiplying the frequency of food consumption and the typical portion size by the nutrient content of each food using the HHHQ-Dietary Analysis Personal Computer System (DIET-SYS; version 4.01) and its accompanying dietary composition database (NCI 1997). Nutrient levels obtained using the HHHQ have been shown to correlate reasonably well with data obtained using 24-hr recall food records and serum nutrient levels (Block et al. 1990; Coates et al. 1991; Hartman et al. 1996). Our *a priori* hypotheses involved protein, folate, zinc, vitamin B_12_, and several of the other nutritional variables that have been linked to arsenic methylation and toxicity in laboratory studies (NRC 1999). However, results for all of the nutritional variables routinely calculated by the DIETSYS program are presented in this article. Selenium has been linked to arsenic methylation in several studies (Christian and Hopenhayn 2004; Hsueh et al. 2003), but we did not assess selenium in this study because of the potentially large inaccuracies in using food frequency questionnaire information to quantify selenium intake (Zhuo et al. 2004).

We calculated the relative proportion of each arsenic species (percent InAs, percent MMA, and percent DMA) by dividing the concentration of each species by the total arsenic concentration, defined as the sum of InAs, MMA, and DMA. Because two to three urine samples were collected from each subject, results from each sample were averaged to obtain a single value for each subject. The intraclass correlation coefficients (ICCs) for the proportions of each metabolite between samples taken at different points in time ranged from 0.45 to 0.68 (Steinmaus et al. 2005). The association of each arsenic species with variables such as age, sex, and smoking history were first assessed using univariate analyses. We also evaluated the association between species proportions and total urinary arsenic. Associations between arsenic dose and methylation patterns have been identified in previous studies, although these generally involve exposures that are much higher than in our study and these associations have typically been small (NRC 1999). The Student *t*-test and the Wilcoxon rank-sum test were used to compare category means. All analyses were initially done separately for cases and controls. However, because we identified no differences between these groups in the relationship between dietary factors and arsenic species in urine, cases and controls are pooled in the results presented here. Arsenic-caused cancer has an estimated latency of ≥ 20 years (NRC 1999). In many of our subjects, their current water source was not the same as their water source ≥ 20 years previously. Because we measured urinary arsenic levels near the time our cancer cases were diagnosed, we did not expect to find a correlation between cancer and the urinary arsenic levels in this study.

Associations between nutrient levels and the proportions of each arsenic species were assessed in two ways. First, the mean proportions of InAs, MMA, and DMA in subjects in the upper and lower quartile of each nutrient variable were compared using the Student *t*-test. Because the intake of most nutrients is strongly related to total calorie intake, we calculated energy-adjusted nutrient levels using the residual method described by Willett and Stampfer (1998). Second, we performed linear regression using the proportion of each arsenic species as the dependent variable and the energy-adjusted nutrient level as the independent variable. This was done with and without the addition of age (continuous), sex, smoking (current vs. noncurrent smoker), and total urinary arsenic (the sum of InAs, MMA, and DMA as a continuous variable) as independent variables. Entering age or total urinary arsenic as categorical rather than continuous variables had no impact on the results. Entering smoking as pack-years or number of cigarettes smoked per day also did not change the results. All data analyses were carried out using the SAS statistical program package (version 8.0e; SAS Institute Inc., Cary, NC).

## Results

In total, 87 subjects agreed to provide urine samples and complete the dietary questionnaire. Table 1 shows the distribution of demographic and lifestyle variables among the study participants. Twenty-two subjects were female (25%), 14 were current smokers (16%), 23 had a history of bladder cancer (26%), and the average age was 68 (range, 21–98 years).

Table 1 also shows the relative proportions of arsenic species and the results of the univariate analyses comparing demographic variables and species proportions. Females excreted a lower percent InAs and percent MMA and a higher percent DMA than did men. Current smokers excreted a higher percent InAs and a lower percent DMA than did former and never-smokers, although these differences were not statistically significant. Increasing age was associated with decreasing percent InAs, but no association was seen between age and percent MMA or percent DMA. The proportion of each arsenic species was similar between cases and controls, and no significant association was seen between total urinary arsenic and the proportion of each arsenic species. Adjusting the total urinary arsenic levels for urine creatinine had no impact on our results.

Table 2 shows the mean level of each nutrient and the mean percent InAs, percent MMA, and percent DMA for the lower and upper quartile of each energy-adjusted nutrient residual. Subjects in the lowest quartile of protein, iron, thiamine, niacin, vitamin B_6_, zinc, and α-carotene intake had a higher mean percent InAs, a higher mean percent MMA, and lower mean percent DMA than subjects in the uppermost quartile of these nutrients, although in some of these comparisons the *p*-value for the differences was > 0.05. For subjects in the lower and upper quartiles of protein intake, respectively, the mean proportions of each arsenic species were 13.1 and 11.4% for percent InAs (*p* = 0.23), 14.6 and 11.6% for percent MMA (*p* = 0.01), and 72.3 and 77.0% for percent DMA (*p* = 0.01). The difference between the median nutrient values for subjects in the upper quartile and subjects in the lower quartile was 25.7 g for protein, 5.64 mg for iron, 0.67 mg for thiamine, 8.34 mg for niacin, 0.58mg for vitamin B_6_, and 545.3 μg for α-carotene. Similar findings were identified when the MMA:DMA ratio was assessed. For example, the MMA:DMA ratio in those in the lower and upper quartiles of protein intake were 0.21 and 0.15 (*p* = 0.008), respectively (data not shown). Clear and consistent threshold patterns were not seen in our analysis. For example, mean percent MMA and percent DMA values for subjects in the two middle quartiles of protein intake were 13.1 and 75.0%, respectively. These are approximately midway between values for subjects in the upper and lower quartiles. In analyses comparing the upper and lower quartiles of nutrient levels that were not adjusted for energy intake, no clear associations were seen between any nutrient and percent InAs, percent MMA, or percent DMA (data not shown).

Table 3 shows the results of the linear regression analysis, adjusted for age, sex, smoking, and total urinary arsenic. Increases in protein intake were associated with decreases in percent MMA [linear regression coefficient (*b*) = −0.075; *p* = 0.02]. This corresponds to an increase of 1.5% in percent MMA for every 20-g decrease in protein intake. High iron and niacin intakes were associated with increases in percent DMA, and increases in oleic acid intake were associated with decreases in percent InAs. Inclusion of age, sex, smoking, and total urinary arsenic in the linear regression model had relatively small impacts on these results. For example, the regression coefficient for protein and percent MMA was −0.084 (*p* = 0.01) in the model that did not include age, sex, smoking, and total urinary arsenic, and −0.075 (*p* = 0.02) in the model that included these variables.

## Discussion

The findings of this study suggest that low intakes of dietary protein, iron, zinc, and niacin lead to a decreased production of DMA and increased levels of MMA in arsenic-exposed individuals. Links between methylation patterns and dietary intake of thiamine, vitamin B_6_, lutein, and α-carotene were also identified in the unadjusted analysis but were less clear after adjustment for age, sex, smoking, and total urinary arsenic levels. As a whole, the results of this study provide some evidence that certain dietary variables can affect arsenic methylation in humans. Although multiple comparisons were performed in this study and some of our findings could be due to chance, several of our results are consistent with those of previous investigations.

The impact of diet on arsenic metabolism and toxicity has been controversial because the risk assessment process used by the U.S. Environmental Protection Agency (EPA) to establish the U.S. drinking water standard for arsenic is based primarily on dose–response information from poorly fed populations in Taiwan (Morales et al. 2000; NRC 2001; U.S. EPA 2001). It has been hypothesized that the Taiwanese populations were particularly susceptible to the health impacts of arsenic as a result of their poor diets, and therefore, the results of studies done in Taiwan may not be relevant to better-fed populations such as those in the United States (Carlson-Lynch et al. 1994; NRC 2001). Although several dietary variables have been mentioned as part of this hypothesis, much of the past debate on this issue was based on whether or not people with low dietary intakes of protein had sufficient amounts of choline, methionine, or cysteine to fully metabolize InAs to DMA (Beck et al. 1995; Brown and Beck 1996; Carlson-Lynch et al. 1994; Engel and Receveur 1993; Mushak and Crocetti 1995, 1996; Slayton et al. 1996). Although the adequacy of the Taiwanese diet is debatable, studies done in experimental animals have shown that severe protein deficiencies can impair arsenic methylation and excretion (Tice et al. 1997; Vahter and Marafante 1987). However, the relevance of these studies to human arsenic exposures was unknown because most species of experimental animals metabolize and excrete arsenic much differently than humans (NRC 1999, 2001; Vahter 1999). Our study is the first to assess the role of dietary protein intake and arsenic methylation in humans, and our findings suggest that, despite these wide inter-species differences, the impacts of protein on arsenic metabolism that have been reported in experimental animals may also occur in human populations.

Protein deficiencies have been linked not only to changes in arsenic methylation but also to increased risks of arsenic-caused adverse effects. In two separate studies in mice, low dietary protein caused increases in DNA hypomethylation and increases in developmental toxicity (Lammon and Hood 2004; Okoji et al. 2002). Several human studies have identified associations between indicators of general malnourishment and the development of arsenic-caused skin lesions, skin cancer, and cardiovascular effects (Chen et al. 1988; Chen et al. 2003a; Guha Mazumder et al. 1998; Hsueh et al. 1995), although the specific role of protein was not addressed in these studies. Only one published human study has investigated the role of protein intake on arsenic-related disease. Mitra et al. (2004) investigated associations between arsenic-caused skin lesions and nutrient intakes, measured using 24-hr dietary recalls, in 238 subjects from West Bengal, India. Elevated odds ratios were seen in subjects with low intakes of calcium [OR = 1.89; 95% confidence interval (CI), 1.04–3.43], fiber (OR = 2.20; 95% CI, 1.015–4.21), and folate (OR = 1.67; 95% CI, 1.87–3.20). In addition, subjects in the lowest quintile of animal protein intake had a skin lesion odds ratio of 1.94 (95% CI, 1.05–3.59) compared with subjects in the highest quintile of animal protein intake. As a whole, the results of these studies, combined with the findings of our investigation, provide a small but emerging body of evidence that low intakes of dietary protein can affect arsenic methylation and may increase in arsenic-associated toxicity.

Although our findings regarding protein are consistent with those of a few other studies, the magnitude of the effect we identified is relatively small compared with the wide inter-individual variability typically seen in arsenic methylation patterns. The differences we identified in percent InAs, percent MMA, and percent DMA between subjects in the upper and lower quartiles of protein intake were 1.7, 3.0, and 4.7%. In comparison, the overall range in percent InAs, percent MMA, and percent DMA in our study population was 29, 23, and 39%, respectively. In an analysis of variance, energy-adjusted protein intake explained only 7.3% of the total variance seen in percent MMA in our subjects. The 3.0% difference in percent MMA we identified between the upper and lower quartile groups of protein intake is of similar magnitude to the impacts identified for some of the other variables most strongly linked to methylation status, including sex and certain genetic polymorphisms (Chiou et al. 1997; Hopenhayn-Rich et al. 1996b). However, studies linking arsenic methylation patterns to increased cancer risks have, to date, not provided sufficient information to estimate dose–response relationships. Thus, the exact impact that these relatively small changes in methylation patterns have on arsenic-caused cancer risks is currently unknown.

In addition to protein, we identified associations between arsenic methylation and iron intake. In the West Bengal study discussed above, the mean daily intake of iron was lower in subjects with arsenic-caused skin lesions than in controls, but this difference was relatively small (13.1 mg in cases and 14.6 mg in controls, *p* = 0.07) (Mitra et al. 2004). In one study, oral administration of iron reduced arsenic-caused DNA damage in mice, although it is unknown whether this effect is related to impacts on arsenic methylation (Poddar et al. 2000). Zinc has been linked to decreased arsenic toxicity in some studies (Milton et al. 2004; NRC 1999; Rabbani et al. 2003) but not in others (Mitra et al. 2004; Shimizu et al. 1998; Wang 1996). In our study, subjects with higher intakes of zinc had lower percent MMA and higher percent DMA, although these results are not statistically significant. We also identified associations between methylation patterns and dietary niacin but are not aware of any animal or human studies that have identified a similar association.

Several other dietary variables that have been directly or indirectly linked to arsenic metabolism in previous animal or in vitro studies, including β-carotene, vitamin E, folate, and vitamin B_12_, were not clearly associated with arsenic methylation patterns in our study (Brouwer et al. 1992; Buchet and Lauwerys 1985; Hsueh et al. 2003). There are several possible reasons why we may have underestimated or missed some associations. One possibility is that certain dietary variables may have substantial impacts only when nutritional deficiencies are severe. In our study, almost all subjects had intakes of protein, iron, vitamin A, thiamine, and other nutrients above U.S. recommended dietary allowance values. In the blackfoot region of Taiwan, where many of the early studies linking ingested arsenic to cancer took place, the mean intake of protein was similar to that of our study subjects (60 g/day in the Taiwanese and 64 g/day in our subjects) (Engel and Receveur 1993; Yang and Blackwell 1961). However, the proportion of subjects in Taiwan with severe deficiencies is unknown, and mean intakes of other variables, such as niacin and zinc, may have been below recommended levels (Engel and Receveur 1993; NRC 1999). In the West Bengal study discussed above, only 44% of subjects had protein intakes above recommended levels (Mitra et al. 2004). Although it is possible that the impacts of diet on arsenic methylation may be greater in populations where nutritional deficiencies are severe, high risks of arsenic-associated cancers and other diseases are not limited to malnourished populations and have been reported in populations where overall nutrition is good (Ferreccio et al. 2000; Hopenhayn-Rich et al. 1996a, 1998; Smith et al. 1998, 2000).

Errors in assessing diet or methylation status could have biased the effect estimates in this study. Although a validated diet questionnaire was used, we asked subjects to provide an estimate of their typical diet over a 1-year period. If methylation patterns depend more on day-today dietary choices than on long-term dietary trends, and subjects changed diets substantially from day to day, the magnitude of any true effects may have been biased. Large intraindividual variability in arsenic methylation patterns could have caused similar bias, although we may have diminished this somewhat by collecting multiple urine samples from each subject and basing methylation status on average values. In measuring both diet and methylation patterns, any misclassification would most likely have been nondifferential and therefore have biased our results toward the null rather than toward spurious associations.

Another explanation for the relatively small impacts we identified in this study is that the dietary variables we assessed may indeed play only a small role in arsenic methylation, and other environmental or genetic factors may have a more predominant role. The *r*^2^ values for the percent MMA and percent DMA regression models including each dietary variable with age, sex, smoking, and total urinary arsenic were all < 0.26 (Table 3), suggesting that these variables explain only a small portion of the total variance seen in percent MMA and percent DMA in our subjects. The results of several studies suggest that inherited genetic traits can have important influences on individual methylation patterns (Chung et al. 2002; Concha et al. 2002; Vahter 1999, 2000, 2002). For example, in a study of 11 families in Chile, the correlation in percent MMA in sibling–sibling pairs, whose genetic makeup is likely very similar, was greater than that in mother–father pairs, who would not necessarily share the same genetic traits (ICC = 0.69, *p* < 0.01 in sibling–sibling pairs; ICC = 0.01, *p* = 0.97 in mother–father pairs) (Chung et al. 2002). In a study of arsenic-exposed residents in Taiwan, subjects with the null genotype of glutathione *S*-transferase M1 had a higher proportion of urinary arsenic in the inorganic form than those with the non-null genotype (regression coefficient = 3.8, SD = 1.9, *p* < 0.05) (Chiou et al. 1997). Other studies have shown that arsenic methylation patterns may vary by ethnicity (Vahter 2000, 2002). Inheritance has also been shown to be a major factor in the individual variation of the activity of several other human methyltransferases (Weinshilboum 1992, 1988).

The trivalent form of MMA was not measured as part of this study. MMA3 is rapidly oxidized to MMA5 in human urine and could not be reliably measured in field investigations at the time this study was done. Several studies have shown that MMA3 is more acutely toxic than other arsenic species (Cullen et al. 1989; Lin et al. 1999, 2001; Mass et al. 2001; Petrick et al. 2000; Styblo et al. 1997, 1999, 2000). However, only a few studies have investigated the presence of MMA3 in nonchelated humans (Del Razo et al. 2001; Mandal et al. 2001; Valenzuela et al. 2005; Wang et al. 2004). Given the high toxicity of MMA3, and the links between total MMA and arsenic-associated cancer risks reported in several investigations (Chen et al. 2003a, 2003b; Del Razo et al. 1997; Hsueh et al. 1997; Maki-Paakkanen et al. 1998; Yu et al. 2000), future studies on MMA3 and its role in human toxicity could add important insights into the mechanisms of arsenic-caused health effects.

In conclusion, the data presented here suggest that dietary protein intake and possibly other nutritional deficiencies can affect arsenic methylation, although the impacts we identified in this well-fed population are small compared with the wide interindividual variability seen in this metabolic process. Additional research on dose–response relationships between arsenic methylation and chronic health effects, as well as further information on the environmental and genetic factors that control arsenic methylation, may help in the identification of susceptible subpopulations and could provide important insights into the carcinogenic mechanisms of this common drinking water contaminant.

## Figures and Tables

**Table 1:** Demographic variables and proportions of arsenic species (mean ± SD).

Variable	No. (%)	Percent InAs	Percent MMA	Percent DMA
All	87 (100)	12.1 ± 4.9	13.1 ± 3.9	74.8 ± 7.0
Sex				
Women	22 (25)	10.3 ± 2.7	10.7 ± 2.4	79.0 ± 3.6
Men	65 (75)	12.7 ± 5.3	13.9 ± 4.0	73.4 ± 7.4
*p*-Value		0.009	< 0.001	< 0.001
Smoking				
Current	14 (16)	14.7 ± 7.5	13.0 ± 5.3	72.3 ± 11.2
Former	47 (54)	11.6 ± 4.1	13.5 ± 3.8	74.9 ± 6.0
Never	26 (30)	11.6 ± 4.0	12.4 ± 3.2	76.0 ± 5.8
*p*-Value		0.15	0.94	0.34
Age (years)				
< 65	24 (28)	13.2 ± 4.7	11.9 ± 2.5	74.9 ± 6.4
65–75	34 (39)	13.0 ± 5.0	13.6 ± 4.5	73.3 ± 8.2
> 75	29 (33)	10.0 ± 4.3	13.4 ± 3.8	76.6 ± 5.9
*R* (*p*-value)^a^		−0.24 (0.03)	0.15 (0.17)	0.08 (0.46)
Urinary arsenic (μg/L)^b^				
< 9.9	29 (33)	12.9 ± 5.4	13.4 ± 4.2	73.8 ± 8.4
9.9–20.3	29 (33)	11.3 ± 3.6	12.5 ± 4.3	76.2 ± 5.8
> 20.3	29 (33)	12.0 ± 5.4	13.4 ± 3.0	74.6 ± 6.7
*R* (*p*-value)^a^		0.05 (0.63)	−0.03 (0.76)	−0.02 (0.87)

a Pearson correlation coefficient and associated p-value.

b Total urinary arsenic was defined as the sum of InAs, MMA, and DMA.

**Table 2:** Mean daily intake of each dietary variable, and the mean proportion of arsenic species in the upper and lower quartiles of each energy-adjusted dietary variable.

		Percent InAs			Percent MMA			Percent DMA		
Nutrient	Nutrient levels (mean ± SD)	Lower quartile	Upper quartile	*p*-Value	Lower quartile	Upper quartile	*p*-Value	Lower quartile	Upper quartile	*p*-Value
Protein (g)	64.1 ± 28.6	13.1	11.4	0.23	14.6	11.6	0.01	72.3	77.0	0.01
Fat (g)	77.3 ± 42.2	13.2	12.5	0.65	12.4	12.4	0.98	74.4	75.1	0.72
Carbohydrates (g)	188.1 ± 81.4	13.4	12.2	0.42	12.7	13.7	0.44	73.9	74.1	0.94
Calcium (mg)	746.8 ± 425.8	11.3	12.1	0.59	13.2	13.6	0.73	75.4	74.2	0.58
Phosphorus (mg)	1107.7 ± 521.1	12.8	10.9	0.25	12.8	12.4	0.72	74.4	76.6	0.29
Iron (mg)	11.6 ± 4.7	14.1	11.1	0.05	14.8	12.5	0.06	71.0	76.4	0.02
Sodium (mg)	2820.4 ± 1487.2	13.1	11.9	0.52	14.2	11.9	0.06	72.7	76.2	0.17
Potassium (mg)	2619.5 ± 971.3	11.8	10.4	0.30	12.6	12.3	0.74	75.5	77.2	0.30
Vitamin A (IU)	7201.8 ± 8269.1	13.4	11.5	0.20	14.0	12.6	0.30	72.6	75.9	0.16
B_1_/thiamine (mg)	1.22 ± 0.57	12.7	11.1	0.27	14.3	11.9	0.05	72.9	76.9	0.06
B_2_/riboflavin (mg)	1.66 ± 0.83	12.3	11.2	0.43	14.2	12.8	0.18	73.5	76.0	0.19
B_3_/niacin (mg)	16.0 ± 6.8	12.7	11.1	0.27	14.8	12.1	0.03	72.5	76.8	0.05
Vitamin C (mg)	114.2 ± 70.1	13.2	12.2	0.57	13.8	12.1	0.17	73.0	75.6	0.25
Saturated fat (g)	27.9 ± 16.3	13.5	11.1	0.18	13.3	12.7	0.59	73.2	75.6	0.24
Oleic acid (g)	27.9 ± 16.1	13.6	12.3	0.41	13.3	12.8	0.66	73.0	74.9	0.44
Linoleic acid (g)	13.7 ± 7.2	12.1	13.1	0.58	13.8	13.3	0.69	74.1	73.6	0.85
Cholesterol (mg)	328.2 ± 243.8	11.5	12.4	0.46	12.3	12.9	0.61	76.2	74.7	0.42
Fiber (g)	11.8 ± 5.2	13.7	11.7	0.19	13.7	13.4	0.79	72.6	74.9	0.30
Folate (μg)	239.2 ± 103.8	12.2	11.6	0.69	13.8	12.3	0.16	74.0	76.1	0.26
Vitamin E (ATE)	8.2 ± 3.5	12.7	13.8	0.49	13.1	12.7	0.75	74.2	73.4	0.77
Zinc (mg)	9.7 ± 4.4	12.9	12.0	0.54	14.1	12.5	0.17	73.0	75.5	0.18
Vitamin B_6_ (mg)	1.28 ± 0.56	12.4	11.9	0.75	14.8	11.8	0.004	72.8	76.3	0.08
Magnesium (mg)	259.5 ± 99.0	11.9	11.5	0.76	13.1	12.1	0.30	74.9	76.4	0.41
α-Carotene (μg)	408.6 ± 1032.0	14.1	10.3	0.02	13.9	12.3	0.13	72.0	77.5	0.02
β-Carotene (μg)	2805.4 ± 3810.7	13.1	12.1	0.47	14.1	12.3	0.20	72.8	75.6	0.23
Lutein (μg)	2152.2 ± 2868.2	10.9	12.4	0.19	14.3	12.1	0.04	74.8	75.6	0.66
Lycopene (μg)	1173.0 ± 1082.8	13.1	12.0	0.51	13.0	12.6	0.69	73.8	75.4	0.51
Retinol (μg)	577.7 ± 350.6	12.7	12.4	0.86	13.6	12.9	0.61	73.7	74.7	0.69
ProA-carotene (μg)	3159.0 ± 4761.5	14.0	11.6	0.15	13.6	11.9	0.13	72.4	76.5	0.08
Cryptoxanthin (μg)	59.5 ± 60.8	13.4	12.5	0.63	13.2	13.7	0.72	73.4	73.7	0.89

ATE, α-tocopherol equivalents.

**Table 3:** Adjusted linear regression coefficients (± SEs) for nutrient levels using percent InAs, percent MMA, and percent DMA as the dependent variables.^a^

	Percent InAs			Percent MMA			Percent DMA		
Nutrient	*b* (SE)	*p*-Value	*R*^2^	*b* (SE)	*p*-Value	*R*^2^	*b* (SE)	*p*-Value	*R*^2^
Protein (g)	−0.012 (0.040)	0.76	0.18	−0.075 (0.031)	0.02	0.20	0.088 (0.057)	0.12	0.21
Fat (g)	−0.045 (0.028)	0.11	0.21	0.011 (0.023)	0.63	0.14	0.034 (0.041)	0.41	0.20
Carbohydrates (g)	−0.011 (0.011)	0.33	0.19	0.003 (0.009)	0.71	0.14	0.008 (0.017)	0.64	0.19
Calcium (mg)	0.0024 (0.0015)	0.10	0.21	−0.0009 (0.0012)	0.48	0.15	−0.0016 (0.0022)	0.47	0.20
Phosphorus (mg)	0.0020 (0.0018)	0.26	0.20	−0.0016 (0.0015)	0.28	0.15	−0.0004 (0.0026)	0.87	0.19
Iron (mg)	−0.32 (0.16)	0.05	0.22	−0.23 (0.13)	0.09	0.17	0.55 (0.23)	0.02	0.24
Sodium (g)	−0.76 (0.69)	0.28	0.20	−0.31 (0.57)	0.58	0.14	1.07 (1.00)	0.28	0.20
Potassium (g)	−0.18 (0.94)	0.84	0.18	−1.21 (0.75)	0.11	0.17	1.39 (1.34)	0.30	0.20
Vitamin A (1,000 IU)	0.038 (0.063)	0.55	0.19	−0.045 (0.051)	0.38	0.15	0.007 (0.091)	0.94	0.19
B_1_/thiamine (mg)	−2.5 (1.6)	0.12	0.21	−1.7 (1.30)	0.18	0.16	4.2 (2.3)	0.07	0.23
B_2_/riboflavin (mg)	0.62 (0.92)	0.50	0.19	−0.91 (0.74)	0.22	0.16	0.29 (1.32)	0.83	0.19
B_3_/niacin (mg)	−0.25 (0.13)	0.05	0.22	−0.20 (0.11)	0.07	0.18	0.45 (0.18)	0.02	0.25
Vitamin C (mg)	−0.0002 (0.0078)	0.98	0.18	−0.0040 (0.0063)	0.53	0.15	0.0042 (0.0112)	0.71	0.19
Saturated fat (g)	−0.088 (0.069)	0.21	0.20	0.049 (0.057)	0.39	0.15	0.039 (0.101)	0.70	0.19
Oleic acid (g)	−0.16 (0.07)	0.02	0.23	0.02 (0.06)	0.75	0.14	0.14 (0.10)	0.17	0.21
Linoleic acid (g)	−0.012 (0.109)	0.91	0.18	0.021 (0.089)	0.81	0.14	−0.009 (0.157)	0.95	0.19
Cholesterol (mg)	0.0009 (0.0033)	0.77	0.18	0.0016 (0.0026)	0.54	0.15	−0.0026 (0.0047)	0.58	0.19
Fiber (g)	−0.045 (0.112)	0.69	0.19	−0.051 (0.091)	0.57	0.15	0.097 (0.160)	0.55	0.20
Folate (μg)	−0.0003 (0.0060)	0.95	0.18	−0.0061 (0.0048)	0.21	0.16	0.0064 (0.0086)	0.46	0.20
Vitamin E (ATE)	0.020 (0.203)	0.92	0.18	−0.079 (0.165)	0.63	0.14	0.059 (0.292)	0.84	0.19
Zinc (mg)	−0.22 (0.22)	0.32	0.19	−0.34 (0.18)	0.06	0.18	0.57 (0.32)	0.08	0.22
Vitamin B_6_ (mg)	0.76 (1.59)	0.63	0.19	−2.04 (1.28)	0.11	0.17	1.27 (2.28)	0.58	0.19
Magnesium (mg)	−0.0005 (0.00760	0.94	0.18	−0.0066 (0.0061)	0.28	0.15	0.0072 (0.0108)	0.52	0.20
α -Carotene (mg)	0.13 (0.50)	0.79	0.18	−0.36 (0.41)	0.38	0.15	0.23 (0.72)	0.75	0.19
β -Carotene (mg)	0.11 (0.130)	0.41	0.19	−0.09 (0.11)	0.40	0.15	−0.02 (0.20)	0.92	0.19
Lutein (mg)	0.28 (0.17)	0.10	0.21	−0.15 (0.14)	0.31	0.15	−0.14 (0.25)	0.58	0.19
Lycopene (mg)	−0.03 (0.48)	0.94	0.18	−0.73 (0.38)	0.06	0.18	0.77 (0.68)	0.26	0.20
Retinol (μg)	0.0019 (0.0017)	0.27	0.20	−0.0011 (0.0014)	0.44	0.15	−0.0008 (0.0024)	0.74	0.19
ProA-carotene (mg)	0.052 (0.109)	0.63	0.19	−0.072 (0.089)	0.42	0.15	−0.019 (0.157)	0.90	0.19
Cryptoxanthin (μg)	−0.0037 (0.0086)	0.67	0.19	0.0054 (0.0070)	0.44	0.15	−0.0017 (0.0124)	0.89	0.19

ATE, α-tocopherol equivalents.

a Adjusted for age, sex, smoking, and urinary total arsenic.
